# Effects of Metacognitive Training on Cognitive Insight in a Sample of Patients with Schizophrenia

**DOI:** 10.3390/ijerph16224541

**Published:** 2019-11-16

**Authors:** Miguel Simón-Expósito, Elena Felipe-Castaño

**Affiliations:** 1 Clinical Psychologist, SESPE, Regional Government of Extremadura, 1003 Cáceres, Spain; miguelfsimon@gmail.com; 2 Lecturer in Psychological Treatment, Evaluation & Personality, University of Extremadura, 1003 Cáceres, Spain

**Keywords:** metacognitive training, cognitive insight, positive symptoms, schizophrenia

## Abstract

Metacognitive training (MCT) is a group intervention that addresses cognitive biases and distortions that could help maintain delusions and hallucinations in people with schizophrenia. This program has proven its effectiveness in reducing the symptoms, but its impact on cognitive insight has scarcely been investigated. Therefore, the aim of the study was to assess the program’s impact on cognitive insight in patients with long-term schizophrenia. A sample of 22 patients with schizophrenia was divided into two groups: one received 16 sessions of MCT (*n* = 11), while the other received the usual treatment (*n* = 11). They were assessed using the Beck Cognitive Insight Scale which measures two components, self-reflection and self-certainty, and the Positive and Negative Syndrome Scale (PANSS). The experimental group showed high levels of adherence, an increase in self-reflection, and a decrease in self-assurance levels as hypothesized. We found statistically significant differences between the control and experimental groups in excitation, hostility, positive symptomatology total score, hallucinatory behavior, and suspicion. In the usual treatment group, a non-significant decrease in positive symptoms was also observed. The findings showed that the implementation of the MCT program in real clinical settings can contribute to an improvement in the metacognitive ability and symptomatology of people with schizophrenia.

## 1. Introduction

Delusions and hallucinations form the central symptomatic nucleus of schizophrenia. Pharmacological intervention does not always have a satisfactory effect on the delirious content or the conviction with which the content is maintained [1]. So, in recent years, research has focused on alterations in the gathering, selection and processing of information that could contribute to the maintenance of delusional thinking [2]. 

Various cognitive biases have been described in people with schizophrenia. One is the tendency to make solid judgments on the basis of scarce evidence which leads patient to make hasty decisions [3]. This dysfunction, called “*jumping to conclusions*” (JTC), has been demonstrably proven, and there is evidence that patients do not recognize it thus increasing the probability of developing delusional ideas [4]. It has also been shown that they fabricate a large quantity of false memories which they believe with great certainty [5].

Other characteristic biases are the tendency to blame others for their own faults while attributing success to themselves [6] and to look for monocausal explanations for what happens to them [7]. Also well documented are the *Theory of Mind* problems within the framework of studies on social cognition [8].

Finally, research has shown the inability of an important number of patients to admit arguments that go against their own conclusions or to recant their own firm beliefs, denominated as “*disconfirmation bias*”, and which also seems to be one of the pillars sustaining the development and maintenance of delusions in persons with schizophrenia [9,10].

In recent years, several metacognitive orientation therapies have been developed in psychosis such as metacognitive reflection and intuition therapy (MERIT) [11] or metacognitive-oriented social skills training (MOSST) [12]. Along the same line, Moritz and Woodward [13] developed the metacognitive training program (MCT) with the main aim of changing the “cognitive infrastructure” of delusional thoughts, increasing the patient’s awareness of distortions, and teaching the patient to reflect critically on them, dealing with the biases that could be the origin of the delusion.

In the last few years, there have been several reviews dealing with the effectiveness of the program in reducing the positive symptomatology and ideation. The most recent [14], which includes 11 high-quality randomized controlled studies, confirms the small to moderate effect of the program on the immediately following measures. This same effect was noted in previous reviews, although a smaller number of studies were included [15,16]. There have also been reports of improvements in cognitive biases [17], and MCT is a safe program, well-accepted by the patients [18].

The MCT aims to promote a greater cognitive flexibility and tries to reduce any excessive confidence in one’s conclusions and, by extension, one’s delusional beliefs. It would thus seem appropriate for improving the so-called cognitive insight [19], a concept defined as the individual’s capacity to evaluate his/her own beliefs and interpretations and to correct them should they be distorted. There are two underlying constructs: self-reflection (SR), a measure of introspection, objectivity, and the capacity to be open to feedback; and self-certainty (SC), which measures the degree to which patients trust their own beliefs and resist correction.

There are few studies that have analyzed the influence of MCT on cognitive insight. Balzan et al. [20], using the individual format of the program, came up with inconclusive results, although they suggested a non-significant improvement in insight based on the increase in SR and the decrease in SC. Lam and colleagues [21], applying the group version, refer to this same improvement due to the increase in the SR, more than the decrease in the SC. Recently, its effectiveness has been reported in initial psychotic episodes with regard to group psycho-education [22].

The aim of our study was to compare the effectiveness of MCT against the usual treatment on cognitive insight in a sample of patients with chronic schizophrenia. As a secondary aim, we evaluated the impact of the MCT on positive symptomatology and examined the program’s degree of applicability in our context. We hypothesized that MCT significantly improves cognitive insight, (e.g., increasing self-reflection and decreasing self-certainty levels) while also contributing to a significant reduction in positive symptomatology.

## 2. Materials and Methods 

The participants were 22 patients, 16 males and 6 females, selected randomly from patients in two residential centers for persons with serious mental disorders (i.e., residential rehabilitation centers). Two of the region’s public residential centers for persons with serious mental disorders, both working under a homogeneous therapeutic regime, were chosen to carry out the study. Patients gain access to the said centers from a single, centralized waiting list that guarantees random assignation of the residents to each center. Thus, the patients were selected from among the 76 residents who fulfilled the established criteria in both centers. One of the centers was chosen as the experimental group and the other as the control group so as to respect the patients’ normal therapeutic environment and for obvious ethical reasons. Then, from among those who fulfilled the criteria, 11 patients were randomly selected from each group. Those patients included were diagnosed with schizophrenia according to the DSM-IV criteria, confirmed by the referring psychiatrist from the mental health team. Patients had to present a clinical stability of at least three months (without modifications in their antipsychotic treatment and no episodes of psychiatric hospitalization during that period). Patients with a prior history of severe brain damage, those who had been in a coma for more than 48 h, those with co-morbid substance dependence, or those who presented intellectual disabilities according to their medical records were all excluded. In order to maximize the relevance of the study in clinical practice, no threshold criterion for PANSS-positive symptoms was defined as an exclusion criterion. Table 1 shows the sociodemographic characteristics of the participants divided into groups.

Each participant was informed verbally and in writing about the objectives of the study, the degree and nature of their participation as well as of the planned assessments. Written, informed consent was obtained from the patients or their tutors in cases of legal incapacitation. The study complied with the ethical norms of the Declaration of Helsinki of 1975, including its revisions, and was approved by the Regional Committee for Research Ethics.

*Beck’s Cognitive Insight Scale (BCIS)* [23]. Spanish adaptation by Gutiérrez-Zotes and colleagues [24]. This is a self-reporting measure consisting of 15 items which use a four-point Likert scale: 0 (*not at all in agreement*) to 3 (*totally in agreement*). It evaluates cognitive insight with two dimensions: self-reflection (SR) and self-certainty (SC). A compound index (CI) can be obtained by subtracting both dimensions. The original version of the instrument presents an internal consistency (Cronbach’s alpha) of *α* = 0.68 for the SR subscale and *α* = 0.60 for the SC subscale. The Spanish adaptation obtained internal consistency values for the SR subscale of *α* = 0.59 and for the SC subscale of *α* = 0.62.

*Positive and Negative Syndrome Scale* (*PANSS)* [25]. Spanish adaptation by Peralta and Cuesta [26]. The PANSS evaluates the major psychiatric symptoms in patients with schizophrenia. It is an expert rating applied by a trained rater. The original version is made up of 30 items grouped into three factors: positive syndrome, negative syndrome, and general psychopathology. The Spanish version presents high indices of internal consistency (Cronbach’s alpha) for the positive and negative scales (*α* = 0.72 and *α* = 0.80, respectively) and moderate indices for general psychopathology (*α* = 0.56).

**Figure 1:**
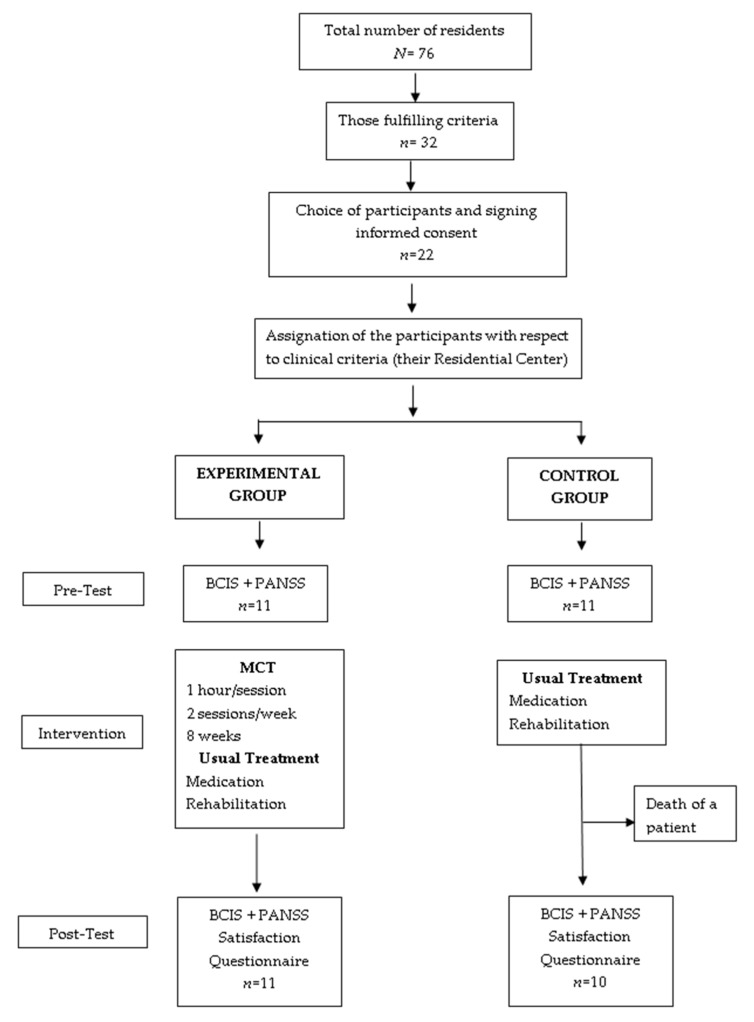
Progress of participants through the stages of the study. MCT: metacognitive training.

The trial procedure is described in Figure 1. All evaluations were carried out by experienced clinical psychologists who were blind to the allocation of the participants. The program was applied by the principal researcher and a psychologist from the residence, both of whom were trained in MCT and had prior experience of its application. The participants followed their usual treatment, which was homogeneous in both centers. No group or individual interventions of a cognitive-behavioral nature were carried out for the time the program lasted. Patients in the control group received MCT once the post-assessment had been completed. None of the patients had previously attended MCT sessions before. The 11 patients of the EMC+TAU condition followed the program till the end (see Figure 1). The average attendance at the sessions was 88% or 96% taking into account justified absences (mainly medical appointments). One patient attended all 16 sessions, five patients attended 15, two patients attended 14, and the other three attended 13 sessions. In addition to the attendance register, a specific register was kept of each session so as to be able to make a qualitative evaluation of a clinical nature concerning the intervention’s impact on the group, to ensure the continuity of the sessions and to register any possible clinical incidences among the participants. No relevant clinical events were observed in the patients during the application of the program.

The MCT program [13] is freely available online. The study used the Spanish version in the group format of 2014 [27]. The program contains two cycles of eight modules each; the participants received two cycles of each module per week—one week: Attribution A and B and then JTC A and B, etc. The mean attendance at the sessions was of 96%. In addition to the registration of adherence, we registered all incidences during each session. All the participants completed the training and no adverse incidents were registered during the sessions.

In analyzing the data, the *t*-test was used to contrast differences between two independent and two dependent groups. The two-way ANOVA was used. The inter-subject factor was the group (experimental or control), while the intra-subject factor was the moment the measurement was taken (pre- or post-test). The analyses were carried out for each measurement variable, for both the intra-subject factor and the interaction with those in the same group. Cohen’s *d* was calculated to estimate the size of the effect of the mean intra-group differences on the experimental and control groups and between the experimental and control groups in the measurement T1. All the analyses were carried out using the SPSS 21 package and bilateral significance tests were also used with a significance level of 5%.

## 3. Results

In the initial measurement, no statistically significant differences were found between the groups in the BCIS indices. However, the experimental group showed a higher psychopathology level, with significant differences in the indices of delusions (*p* = 0.040), hallucinatory behavior (*p* = 0.013), excitation (*p* = 0.030), suspicion (*p* = 0.047), hostility (*p* = 0.019), and in the total score of the positive symptoms subscale (*p* = 0.019).

After applying the MCT program (see Table 2), an average increase was observed in the experimental group of the SR of the BCIS with respect to the same index in the baseline (*d* = 0.43), a decrease in the SC (*d* = 0.50), and an increase in the CI (*d* = 0.63). The size of the average effect, through Cohen’s *d* test, was within the normal range, while it was practically stable in the control group, where the size of the effect was, to all intents and purposes, insignificant. These differences did not become significant, although in the case of the CI, it did come close to significance (*p* = 0.063). 

Once the variance analysis was carried out for each variable and the interaction with the group to which it pertains, taking into account the intra-subject factor and the interaction with the group, no differences were found between the experimental and control groups nor between the pre- and post-test measurements in any of the three indices. The interaction among the group and the average pre- and post-test did not turn out to be significant either (see Table 2).

In the experimental group, we found a decrease in the average scores for all the items of the positive subscale of the PANSS. The analysis of the size of the effect of these changes in the experimental group, measured using Cohen’s *d* test, oscillated between small, in the case of delusions (*d* = 0.27), suspicion (*d* = 0.23), and total score in the positive subscale of the PANSS (*d* = 0.22), and medium in the case of excitation (*d* = 0.40).

We found statistically significant differences between the control and experimental groups in excitation (*p* = 0.043), hostility (*p* = 0.040), and total score for positive symptomatology (*p* = 0.035) across time (see Table 2). We also found these differences in hallucinatory behavior (*p* = 0.003) and suspicion (*p* = 0.026).

As for the interaction between the group and the pre- and post-test scores, we found significant differences in delusions (*p* = 0.050), excitation (*p* = 0.031), hostility (*p* = 0.014), and the total score for positive symptomatology (*p* = 0.032) (see Table 2).

## 4. Discussion

The results obtained did not allow us to confirm the hypothesis, as the differences found were not significant. However, the results do point in the direction set out in the hypothesis, as there was an increase in the average SR score of the experimental group, a decrease in the SC score, and an increase in the CI of almost three points, while the scores remained practically the same, without changes, in the control group. The size of the effect in the experimental group clearly demonstrates this tendency, showing average effect sizes.

The reason for the absence of statistical significance can be found in the fact that, in the data analysis, we took into account the prior level of delirious ideation which was high in the experimental group and which may have had a vital influence on the results as can be deduced from the statistical analysis when this variable is taken into account.

Nevertheless, only Lam and colleagues [21] achieved significant increases in the SR and CI indices and a non-significant decrease in the SC. Other authors [20,28] found no significant differences, and the modification that was registered could even be said to be negligible [29]. Nevertheless, these studies differ from the sample and criteria used in this current work due to the application of the MCT program in its individual version and only partially [20] using younger samples [29] or not incorporating the prior delusional conviction in the analysis [21]. In fact, in our study, the decrease found was almost double in the SC indices with effect sizes that also almost double those of the latter study above with similar increases in the CI.

A decrease in the SC levels supposes casting doubt on one’s own convictions, so a high level of delusion contributes to making this task more difficult. Thus, some authors point out that MCT may not affect patients with moderate to serious delusions [28], and they establish high levels of delusional ideation as a criterion [29] or they include only mild delusions [30]. In our case, the inclusion of these patients had no influence on the development of the sessions, but it seems clear that it did limit the efficacy of the program. Nevertheless, the size of the reduction in the levels of conviction in one’s own beliefs (SC) and the increase in openness to looking for information (SR) supposes a certain impact on the increase in metacognitive awareness, in spite of the deep-rooted nature of the delusional ideation.

It is significant that the patients in the control group, who had higher levels of positive symptomatology, also experienced a greater, more positive impact in the two subscales of cognitive insight. We could speculate with the theory that this impact was connected to the reduction in symptomatology which would be in line with the relationship found among both variables and the influence that the program could have on the reduction in cognitive bias such as that of coming to hurried conclusions [31]. Nevertheless, we found no significant correlation in our sample among these variables in either of the two groups, neither before or after applying the MCT thus we cannot state categorically what actually was the cause of this influence. 

Concerning the impact of MCT on the reduction in the positive symptomatology, the results allowed us to confirm that the program had a significant positive impact on the decrease in the levels of delusional symptomatology, excitation, hostility, and total positive symptomatology, although the size of this effect was small [14].

Several facts may have contributed to the lack of significant decreases. On the one hand, there is the very characteristics of the sample itself, which was made up of patients with chronic symptoms of schizophrenia, serious problems in their daily functioning, a higher average age than the patients from previous studies, and a time-scale for the evolution of their illness which was only comparable to those of patients from one other recent study [17]. This prolonged evolution of the illness, together with the high average age, could contribute to the increase in the cognitive deficits present in general functioning. This, in turn, could influence the effectiveness of an educational program such as that of the MCT. Such is the opinion of some reviews of transversal studies on cognitive deficits in older people with schizophrenia. They indicate that, besides the individual neurocognitive deficits, there are also deficits in global cognition and certain variables, such as age, being male, having a low educational level, the presence of more symptoms, greater institutionalization, and a longer illness, which all contribute to an intensification of the deterioration [32] and which could support this hypothesis.

Thus, the prior incorporation of patients in a cognitive rehabilitation program oriented towards improving their functioning in such areas as concentration, memory or executive functions could be interesting [11] and can be a key determinant of their quality of life [33].

The inclusion in the study of subjects with average and high scores on the delusional scale in the experimental group, scores that were significantly different from those in the control group, to our understanding, may have influenced the impact of the program on the positive symptomatology, as it contributes to the distraction of the group [29]. Within the framework of a group format, this participation has a greater influence. In fact, Moritz and colleagues et al. [34] report significant decreases in the positive subscale of the PANSS using a combination of the group and individual versions of the MCT program. In this sense, an interesting line of work would be to analyze the impact with respect to the severity of the delusions or even the very absence of any delusional symptomatology, in line with the work of Moritz et al. [35] on variables that moderate the impact of the program.

One interesting aspect that must be taken into account is the number of sessions applied. In our study, two series of eight sessions each were carried out to complete both versions of the program which may have had an effect on the reduction in the higher symptomatology than that observed in other studies using only eight sessions. In fact, Briki [36] obtained similar results to ours using 16 sessions in a sample of patients with a very similar profile and high levels of symptomatology. To our understanding, this opens up an interesting line of research into the effect of the intensity of the intervention on the levels of symptomatology. 

Finally, we should point out the high level of program completion on the part of the patients, which coincided with all previous studies [21] and the value of the program’s applicability in a clinical context if we take into account the fact that the program deals with delusional conviction. In addition to the therapeutic benefits inherent to any group intervention, the format has permitted us to make a psycho-educational intervention using fewer resources. Furthermore, in this sense, the clinical experience gained following the application of the program indicates that the attendance of the patients has laid the foundations for a later intervention of an individual nature, oriented towards the specific symptomatology which appears to be much more effective. It would therefore be a positive step to incorporate the MCT with interventions with a metacognitive orientation, such as the already mentioned MERIT, for higher level metacognitive processes, or MOSST for social functioning. The creators of the MCT have, themselves, put forward such a view [37]. 

## 5. Limitations

We are aware of the study’s limitations derived from the clinical context in which it was developed, such as the reduced sample size, its representativity, and the difficulties of randomization. We have already mentioned the use of self-application measures, such as the BCIS, as they can have certain limitations in subjects with an altered capacity for self-reflection. The use of the PANSS as a scale to measure the delusional and hallucinatory symptomatology could also suppose a limitation in the evaluation, it being less sensitive to changes in comparison to other scales, such as the Psychotic Symptom Rating Scales (PSYRATS) [38]. On the other hand, we have not taken into account the effect of medication as a variable that could influence the patients’ cognitive deficits and condition on following the program, for which it would be necessary to control when considering the pairing of the subjects in the base line measurement. 

Finally, although both centers used the same intervention programs, we are also conscious of the fact that not all the possible variables relating to the intervention in the control group were completely controlled which may have introduced a certain bias into the results.

## 6. Conclusions

The MCT had a significant positive impact on the decrease in the levels of delusional symptomatology, excitation, hostility, and total positive symptomatology, although the size of this effect was small.

The findings of our study are relevant for clinical practice, as they suggest the efficacy and the value of the MCT’s applicability in a clinical context. Future research should focus on the previously presented limitations.

## Figures and Tables

**Table 1:** Participants’ sociodemographic and clinical characteristics. Differences between the experimental and control groups.

Participant´s Characteristics	Experimental (*n* = 11)	Control (*n* = 11)	*t*	
	M (SD)	M (SD)		Significance
**Age**	42.82 (7.50)	47.27 (12.63)	−1.422	0.162
Years of study (schooling)	10.36 (4.01)	11.82 (3.14)	−1.340	0.187
Age at which illness began	21.27 (4.41)	22.91 (5.89)	−1.043	0.303
Duration of illness (years)	21.55 (8.26)	24.36 (11.48)	−0.934	0.355

**Table 2:** Differences in the averages between pre- and post-test in the experimental and control groups. Two-way ANOVA. Cohen’s *d* from the MCT.

	Experimental (*n* = 11)					Control (*n* = 10)					Two-Way ANOVA		
	Pre-Test	Post-Test				Pre-Test	Post-Test						
Scales	M (SD)	M (SD)	*t*	Significance	*d*	M (SD)	M (SD)	*t*	Significance	*d*	*F*	Significance	*Partial Eta Squared*
SR	13.27 (3.74)	14.91 (3.96)	−1.292	0.226	0.43	14.20 (4.98)	14.30 (4.83)	−0.079	0.938	0.02	0.735	0.402	0.037
SC	9.00 (2.83)	7.73 (2.19)	1.272	0.232	0.50	9.50 (3.03)	9.00 (3.68)	1.246	0.244	0.15	0.477	0.498	0.024
CI	4.27 (4.82)	7.18 (4.38)	−2.091	0.063	0.63	4.70 (7.11)	5.30 (7.19)	−0.458	0.658	0.01	1.445	0.244	0.071
DEL	3.82 (1.94)	3.27 (2.15)	2.206	0.052	0.27	2.00 (1.63)	2.00 (1.63)	−1.000	0.343	0.00	4.402	0.050	0.188
DISOR	2.64 (1.69)	2.45 (1.81)	1.491	0.167	0.11	2.30 (1.34)	2.40 (1.56)	−1.000	0.343	0.03	3.116	0.094	0.141
HALL	2.82 (1.47)	2.64 (1.63)	0.803	0.441	0.11	1.10 (0.32)	1.10 (0.32)	−1.000	0.343	0.00	0.584	0.454	0.030
EXC	2.73 (1.10)	2.27 (1.19)	2.193	0.053	0.40	1.40 (0.96)	1.50 (1.27)	0.000	1.000	0.09	5.439	0.031	0.223
GRAND	2.18 (1.89)	2.18 (1.89)	0.000	01.00	0.00	1.40 (1.26)	1.50 (1.27)	−2.449	0.037	0.08	0.343	0.565	0.018
SUSP	3.27 (1.35)	3.00 (1.00)	0.760	0.465	0.23	2.00 (1.05)	2.00 (1.25)	−1.413	0.191	0.00	0.457	0.507	0.024
HOST	2.36 (1.03)	2.27 (1.01)	1.000	0.341	0.01	1.30 (0.67)	1.70 (0.67)	−1.500	0.168	0.59	7.248	0.014	0.276
TPPANS	20.00(8.09)	18.09(9.44)	1.955	0.079	0.22	11.50 (4.99)	12.20 (5.79)	−1.647	0.134	0.05	5.334	0.032	0.219

Note: SR: scale of self-reflection of the Beck Cognitive Insight Scale (BCIS); SC: scale of self-certainty of the BCIS; CI: compound index of the BCIS; DEL: delusions; DISOR: disorganization; HALL: hallucinatory behavior; EXC: excitation; GRAND: grandiosity; SUSP: suspicion; HOST: hostility; TPPANS: total score the Positive and Negative Syndrome Scale post-test.
